# Targeted Delivery of TrkB Receptor to Phrenic Motoneurons Enhances Functional Recovery of Rhythmic Phrenic Activity after Cervical Spinal Hemisection

**DOI:** 10.1371/journal.pone.0064755

**Published:** 2013-05-28

**Authors:** Heather M. Gransee, Wen-Zhi Zhan, Gary C. Sieck, Carlos B. Mantilla

**Affiliations:** 1 Department of Physiology and Biomedical Engineering, Mayo Clinic, Rochester, Minnesota, United States of America; 2 Department of Anesthesiology, Mayo Clinic, Rochester, Minnesota, United States of America; Inserm, France

## Abstract

Progressive recovery of rhythmic phrenic activity occurs over time after a spinal cord hemisection involving unilateral transection of anterolateral funiculi at C_2_ (SH). Brain-derived neurotrophic factor (BDNF) acting through its full-length tropomyosin related kinase receptor subtype B (TrkB.FL) contributes to neuroplasticity after spinal cord injury, but the specific cellular substrates remain unclear. We hypothesized that selectively targeting increased TrkB.FL expression to phrenic motoneurons would be sufficient to enhance recovery of rhythmic phrenic activity after SH. Several adeno-associated virus (AAV) serotypes expressing GFP were screened to determine specificity for phrenic motoneuron transduction via intrapleural injection in adult rats. GFP expression was present in the cervical spinal cord 3 weeks after treatment with AAV serotypes 7, 8, and 9, but not with AAV2, 6, or rhesus-10. Overall, AAV7 produced the most consistent GFP expression in phrenic motoneurons. SH was performed 3 weeks after intrapleural injection of AAV7 expressing human TrkB.FL-FLAG or saline. Delivery of TrkB.FL-FLAG to phrenic motoneurons was confirmed by FLAG protein expression in the phrenic motor nucleus and human TrkB.FL mRNA expression in microdissected phrenic motoneurons. In all SH rats, absence of ipsilateral diaphragm EMG activity was confirmed at 3 days post-SH, verifying complete interruption of ipsilateral descending drive to phrenic motoneurons. At 14 days post-SH, all AAV7-TrkB.FL treated rats (n = 11) displayed recovery of ipsilateral diaphragm EMG activity compared to 3 out of 8 untreated SH rats (p<0.01). During eupnea, AAV7-TrkB.FL treated rats exhibited 73±7% of pre-SH root mean squared EMG vs. only 31±11% in untreated SH rats displaying recovery (p<0.01). This study provides direct evidence that increased TrkB.FL expression in phrenic motoneurons is sufficient to enhance recovery of ipsilateral rhythmic phrenic activity after SH, indicating that selectively targeting gene expression in spared motoneurons below the level of spinal cord injury may promote functional recovery.

## Introduction

Most spinal cord injuries (SCI) are incomplete, with some sparing of spinal cord pathways. A well-established model of incomplete SCI, unilateral spinal cord hemisection at C_2_ (SH), removes premotor drive to phrenic motoneurons, paralyzing the ipsilateral diaphragm muscle [1], [2], [3], [4], [5], [6], [7], [8]. Following SH, there is spontaneous recovery of ipsilateral rhythmic phrenic activity due to strengthening of latent contralateral excitatory premotor input to phrenic motoneurons [9], [10], [11], [12], [13], [14]. Although this spontaneous neuroplasticity results in recovery of some ipsilateral diaphragm function after SH, significant impairments remain [7], [10], [15].

Neuroplasticity associated with strengthening of spared synaptic inputs may involve changes in: 1) presynaptic input to phrenic motoneurons; and 2) postsynaptic changes in the excitability of phrenic motoneurons. There is much evidence suggesting that brain-derived neurotrophic factor (BDNF) and its high affinity tropomyosin-related kinase receptor (TrkB) play an important role in synaptic plasticity [16], [17], [18], [19], [20], [21]. BDNF signaling via full-length TrkB (TrkB.FL) likely enhances phrenic motoneuron excitability through serotonergic [22] and glutamatergic signaling [16], [23]. Expression of serotonergic and glutamatergic receptors within phrenic motoneurons increases following SH [6], [24], and the time course of changes generally corresponds with onset of spontaneous recovery of rhythmic phrenic activity. Unfortunately, treatment with exogenous intrathecal neurotrophins is linked to significant adverse effects related to autonomic and sensory (pain) pathways, which prevent it from being used therapeutically [25], [26], [27]. This study explores a novel, targeted approach to increase BDNF/TrkB.FL signaling in phrenic motoneurons that may help avoid undesirable off-target effects of BDNF treatment. We hypothesized that selectively targeting increased TrkB.FL expression to phrenic motoneurons would be sufficient to enhance recovery of rhythmic phrenic activity after SH. Our results demonstrate that the intrapleural delivery technique [28] combined with the selective transduction of phrenic motoneurons by AAV7 provides a novel method of targeting gene expression to phrenic motoneurons, thereby promoting recovery of ipsilateral diaphragm EMG activity after SH.

## Materials and Methods

### Experimental Animals

Adult male Sprague-Dawley rats (Harlan, Indianapolis, IN; initial body weight 220–300 g) were used in these experiments. All procedures were approved by the Institutional Animal Care and Use Committee at Mayo Clinic (Protocol #A51711). Animals were anesthetized with an intramuscular injection of ketamine (90 mg/kg) and xylazine (10 mg/kg) for all surgical procedures and experimental measurements.

### Phrenic Motoneuron Transduction with AAV Vectors

For AAV optimization experiments, AAV vectors encoding GFP under a CMV promoter (AAV.CMV.PI.EGFP.WPRE.bGH) were obtained from the Vector Core at University of Pennsylvania, courtesy of Dr. Julie Johnston. Six different AAV serotypes were tested (2, 6, 7, 8, 9, rhesus-10) using a dose of 2×10^11^ genome copies. AAV was administered via intrapleural injection as previously described [28]. Briefly, AAV was injected using a Hamilton syringe into the pleural space of the right and left sides of the chest between the 7th and 8th ribs (50 µl/side). Control animals were injected with physiological saline (50 µl/side). Animals were euthanized at 3 or 10 weeks after AAV injection.

For AAV-TrkB.FL experiments, rats were used in 5 groups: untreated control (n = 3), AAV7-TrkB.FL treated control (n = 6), untreated SH (n = 8), AAV7-TrkB.FL treated SH (n = 11), and.AAV9-TrkB.FL treated SH (n = 3). AAV7 and AAV9 vectors encoding human TrkB.FL-FLAG under a CMV promoter (AAV7.CMV.Flag-TrkB.WPRE.bGH) were obtained from the Vector Core at University of Pennsylvania. At 21 days prior to SH, rats were given a bilateral intrapleural injection of the AAV7 or AAV9 vector (50 µl; 1×10^11^ genome copies/side).

### Spinal Cord Hemisection (SH)

As previously reported [2], [3], [5], [6], [8], a dorsal C_2_ laminectomy was performed and the right anterolateral cord was transected at C_2_ with a microknife. The injury involved only lateral and ventral funiculi and preserved the dorsal funiculus. The C_2_ SH injury results in prolonged inactivity of the ipsilateral diaphragm muscle [9], [10], [29], although some recovery occurs over time [8], [11], [30]. Animals were administered acetaminophen orally for the first 3 days and buprenorphine intramuscularly as needed. Animals were euthanized at 14 days post-SH (SH 14D).

### Chronic Diaphragm EMG Recordings

Diaphragm EMG activity (presence vs. absence) and root mean squared (RMS) EMG amplitude were chronically monitored as indices of functional recovery ipsilateral to SH, as previously described [31], [32], [33], [34]. At 3 days prior to SH, two pairs of electrode wires (insulated stainless steel, AS631, Cooner Wire Inc., Chatsworth, CA) were stripped about 3 mm at the tip, implanted into the left and right mid-costal hemidiaphragms, and secured such that the uninsulated portion remained within the diaphragm. The wires were tunneled and externalized in the dorsum of the animal.

Eupneic diaphragm EMG activity in anesthetized rats was measured at the time of SH surgery to verify absence of ipsilateral activity, as well as at 3 days post-SH (SH 3D) to confirm complete interruption of descending drive to phrenic motoneurons ipsilateral to SH. EMG was also measured at 7 days post-SH (SH 7D) and SH 14D to determine recovery of rhythmic ipsilateral activity. EMG signals from each pair of electrodes were amplified (2000x) and band-pass filtered between 20–1000 Hz. Signals were digitized with a data acquisition board at a sampling frequency of 2 kHz and recorded using LabView (National Instruments, Austin, TX). Diaphragm RMS EMG amplitude were calculated with a moving window of 50 ms [33], [35]. The criteria for functional recovery classification included: 1) rhythmic (i.e., in phase with the contralateral side) and periodic (i.e., occurring on at least 90% of eupneic bursts) diaphragm EMG signal; 2) diaphragm EMG signal reflecting activation of more than one motor unit; and 3) diaphragm RMS EMG amplitude at least 10% of the pre-SH amplitude.

### Retrograde Labeling of Phrenic Motoneurons

Intrapleural injection of cholera toxin subunit B (CTB) was used to retrogradely label phrenic motoneurons in immunohistochemical and laser capture microdissection experiments. This technique was previously validated for this purpose and labels only phrenic motoneurons in the cervical spinal cord [28]. Briefly, 20 µl of a 0.2% solution of either Alexa Fluor 488-conjugated CTB (Molecular Probes; Life Technologies, Grand Island, NY) or unconjugated CTB (List Biological Laboratories, Campbell, CA) was injected using a Hamilton syringe into the pleural space of the right and left sides of the chest between the 7th and 8th ribs at 3 days before the terminal experiment.

### Immunohistochemistry and Confocal Microscopy

At 3 or 10 weeks after injection with AAV expressing GFP, animals were anesthetized and euthanized by exsanguination following administration of heparin. The cervical spinal cord (C_2_–C_7_) was then fixed by transcardial perfusion with 4% paraformaldehyde. Spinal cord segments were post-fixed in 4% paraformaldehyde in phosphate-buffered saline (PBS; pH 7.4) overnight, and then transferred to 24% sucrose in PBS for 24–72 hours prior to cryosectioning. A Reichert–Jung Frigocut cryostat (Reichert Microscope Services, Depew, NY) was used to cut the samples in 50 µm-thick longitudinal sections.

Spinal cord sections were blocked with 10% Donkey Serum in 0.3% Triton TBS. CTB was detected using a CTB antibody (List Biological Laboratories; goat polyclonal, #703), and GFP fluorescence was enhanced using a GFP antibody (Abcam, Cambridge, MA; rabbit polyclonal, ab6556), followed by secondary antibodies DyLight 649-conjugated anti-goat and Cy3-conjugated anti-rabbit, respectively (Jackson Immunoresearch, West Grove, PA). Tissue sections were mounted on slides, dehydrated in graded alcohols and xylene, and coverslipped with DPX mountant (Fluka, Sigma–Aldrich, St. Louis, MO).

Spinal cord sections were imaged using an Olympus FluoView 300 laser scanning confocal microscope (Olympus America Inc., Melville, NY) mounted on an upright Olympus BX50WI microscope with Argon (488 nm), HeNe (543 nm) and HeNe (633 nm) lasers. Simultaneous imaging was performed using a 540 nm dichroic mirror and emission filters (495–535 nm and 605 nm for Cy3 and DyLight 649, respectively). Fluorescently-labeled phrenic motoneurons were imaged with a 20× oil immersion lens (NA 0.8), and three-dimensional image stacks were collected in an 800×600 array (pixel dimensions: 1 µm×1 µm) with a 1.0 µm step size. Laser intensity, confocal aperture, and photomultiplier gain were kept fixed across samples. Optical slices containing the mid-nuclear region of a CTB-labeled phrenic motoneuron were identified and used to quantify the number of phrenic motoneurons. A phrenic motoneuron was considered GFP immunoreactive if the soma was labeled by GFP or if GFP-labeled dendrites could be traced to the soma.

Maximal projection images for each channel were made from the confocal image stacks using Metamorph Imaging Software version 7.6 (Universal Imaging Corporation, Downingtown, PA) and exported into Adobe Photoshop (version 7.0). GFP images were changed to grayscale and inverted. Brightness and contrast levels were adjusted linearly as needed. For all primary/secondary antibody pairs, additional studies not including the primary antibody (blank) or using tissues from saline-injected animals (not expressing GFP or CTB) were conducted to confirm the specificity of immunostaining, as in previous studies [28], [36]. These sections were processed in parallel for all immunohistochemical reactions and animals.

### Protein Analyses

Measurement of FLAG expression was used to document AAV-mediated transduction of motoneurons in the cervical spinal cord. At 5 weeks after treatment with AAV7-TrkB.FL-FLAG or no treatment, the cervical spinal cord was excised, and the ventral horn region (in the anterior part of each lateral half of the spinal cord, containing phrenic motoneurons) was isolated and frozen in liquid nitrogen. The ventral horn was homogenized in lysis buffer (Cell Signaling Technology, Beverly, MA) with complete mini protease inhibitor (Roche, Indianapolis, IN). The homogenate was centrifuged, and protein concentration was determined using the Bio-Rad DC protein assay (Bio-Rad Laboratories, Hercules, CA). Samples were diluted 1∶1 in Laemmli buffer (Bio-Rad), electrophoretically separated under denaturing conditions on 10% SDS-PAGE Criterion gels (Bio-Rad), and transferred to polyvinylidene difluoride membranes (Bio-Rad). Membranes were blocked in Tris-buffered saline (TBS; pH 7.5) with 5% milk and 0.1% Tween-20 (Sigma-Aldrich), followed by an overnight incubation with primary antibodies for FLAG (Sigma-Aldrich; mouse monoclonal, F1804) and actin (Sigma-Aldrich; rabbit polyclonal, A2066) diluted in TBS containing 5% milk and 0.1% Tween-20. Membranes were incubated with appropriate horseradish peroxidase-conjugated secondary antibodies (Santa Cruz Biotechnology, Santa Cruz, CA), and immunodetection was performed using enhanced chemiluminescence (Pierce Biotechnology, Rockford, IL). Images were obtained with Kodak MM4000 Image Station software (Kodak Molecular Imaging Systems, New Haven, CT).

### Laser Capture Microdissection (LCM) of Phrenic Motoneurons

Phrenic motoneurons were individually captured to measure rat and human TrkB receptor mRNA expression. LCM of phrenic motoneurons pre-labeled with Alexa Fluor 488-conjugated CTB was performed as previously described [6]. Briefly, the cervical spinal cord (C_2_–C_7_) was excised from uninjured rats with or without AAV7-TrkB.FL treatment and immediately frozen in liquid nitrogen under RNase-free conditions. Longitudinal spinal cord sections (10 µm thick) were placed on pre-chilled slides, dehydrated in graded alcohols and xylene. An Arcturus^XT^ LCM microdissection system was used to visualize individual, retrogradely-labeled phrenic motoneurons under direct epifluorescence illumination and then microdissect them onto Capsure HS LCM caps (Arcturus LCM, Applied Biosystems, Life Technologies Corp., Carlsbad, CA). Approximately four caps were obtained from each side of the spinal cord for each animal. Caps were stored at −80°C until RNA extraction.

### Real-time RT-PCR

Total RNA was extracted from microdissection caps using RNeasy Micro kit (Qiagen Inc., Valencia, CA) following the manufacturer’s protocol. The RNA from all caps from each animal was pooled. Total RNA was reverse transcribed using the Transcriptor First Strand cDNA Synthesis kit (Roche Applied Science, Indianapolis, IN) following the manufacturer’s protocol and as previously described in detail [6]. Briefly, sample RNA, anchored-oligo(dT)18 primer, random hexamer primer, and RNase free sterile water were incubated at 65°C for 10 min and chilled on ice for 1 min. A mix with 1× Transcriptor reverse transcriptase reaction buffer, Protector RNase inhibitor, dNTP, and Transcriptor reverse transcriptase was added to each sample and incubated at room temperature for 10 min followed by 20 min at 55°C. Samples were heated to 85°C for 5 min and placed on ice. All reverse transcription reactions were done in duplicate for each sample.

For quantitative analyses of human and rat TrkB mRNA transcripts, reverse transcription reactions were sent to the Medical Genome Facility Gene Expression Core at Mayo Clinic. The Platinum SYBR Green qPCR Mix with UDG (Invitrogen, Life Technologies, Grand Island, NY) was used, and the reverse transcription reaction and primer pairs were added to this reaction mix. Ribosomal protein S16 (RPS16) was used as a reference gene. Amplification and quantitation of mRNA was performed on an AB 7900 HT qPCR machine (Applied Biosystems). The thermal cycling conditions were as follows: 50°C for 2 min; 95°C for 2 min; 40 cycles of 95°C for 15 s followed by 60°C for 60 s. Products were then heated with a melting curve protocol (95°C for 15 s; 60°C for 15 s; 95°C for 15 s at a 2% ramp rate) while measuring fluorescence, yielding a melting curve as previously described [6]. All PCR reactions were performed in duplicate for each reverse transcription reaction.

### Statistical Analyses

All statistical evaluations were performed using standard statistical software (JMP 8.0, SAS Institute Inc.). The proportions of animals displaying functional recovery were compared across groups using Pearson’s chi-square test. Diaphragm RMS EMG amplitude was normalized to the eupneic value before SH for the same animal and differences between treatment groups were examined using one-way analysis of variance. Statistical significance was established at the 0.05 level. All experimental data are presented as mean ± SE, unless otherwise specified.

## Results

### Retrograde Transport of AAV to Phrenic Motoneurons

Animals were injected intrapleurally with AAV2, 6, 7, 8, 9, or rhesus-10 encoding GFP to evaluate selective transduction of phrenic motoneurons by the different AAV serotypes. No adverse effects of the AAV injection were evident for any serotype: all animals displayed normal weight gain and grossly intact behavior. Transduction of phrenic motoneurons was determined by GFP co-localization with CTB in retrogradely-labeled motoneurons. Retrograde labeling with CTB was robust, allowing individual visualization of phrenic motoneurons, as in previous studies [6], [28], [36], [37], [38].

**Figure 1:**
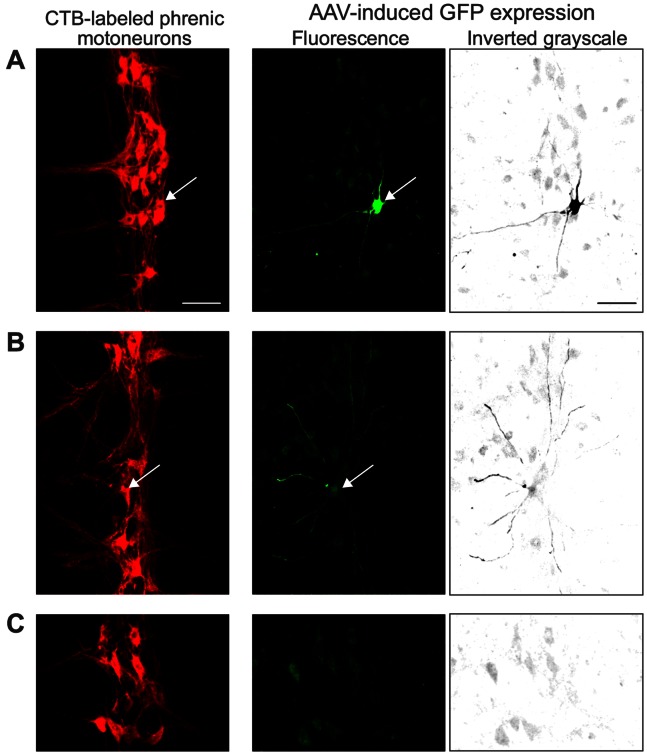
Retrograde delivery of adeno-associated virus (AAV) expressing GFP (AAV-GFP) to phrenic motoneurons by intrapleural injection. Representative confocal photomicrographs of retrogradely-labeled phrenic motoneurons were used to determine GFP co-localization at individual motoneurons. Images shown are maximum intensity projections of confocal slices showing a longitudinal section at ∼C3–4 (orientation: rostral, top; medial, left)., with side-by-side pictures showing the 2 fluorescence channels from the same projection – cholera toxin subunit B-labeled phrenic motoneurons (red) and GFP expression (green). GFP expression was also converted to grayscale and inverted to facilitate visualization (black). A) AAV7-GFP resulted in selective GFP expression in phrenic motoneurons in all treated animals at 3 weeks (n = 6) and 10 weeks (n = 1) after ipsilateral injection. B) AAV7-GFP also resulted in extensive labeling of phrenic motoneuron dendrites but the cell somata were not always identifiable in the same or neighboring spinal cord sections, likely due to GFP leaking after paraformaldehyde fixation, as seemingly happens with eGFP expression. C) Lack of GFP immunoreactivity in the phrenic motoneuron pool 3 weeks after intrapleural saline treatment (n = 6). Images were acquired such that background autofluorescence in the GFP channel was allowed in order to maximize visualization of motoneuron dendrites. Bar, 100 µm in A–C.

Intrapleural injection of AAV7, 8, and 9 resulted in GFP expression in the cervical spinal cord at 3 or 10 weeks. All rats injected with AAV7 consistently displayed GFP expression in both dendrites and soma of phrenic motoneurons at 3 weeks (n = 6; Figure 1). GFP expression persisted through 10 weeks after injection (n = 1). Importantly, GFP expression after AAV7 transduction was detected only in phrenic motoneurons, and was present throughout the entire phrenic motoneuron pool rather than limited to a specific segment. Phrenic motoneuron morphology was not visibly altered. Consistent with previous studies [6], [28], 147±30 labeled phrenic motoneurons where the cell body was completely contained within serial 50 µm longitudinal sections of the cervical spinal cord (i.e., containing the mid-nuclear region) were visualized per animal (Figure 1A). A total of 98 GFP immunoreactive phrenic motoneurons were identified out of 884 motoneurons labeled by CTB in six rats treated with intrapleural AAV7-GFP. Thus, overall transduction efficiency of AAV7 (calculated as the ratio of GFP immunoreactive phrenic motoneurons to the total number of motoneurons) would be at least 11%. This measurement is an underestimate of the actual transduction efficiency given that extensive labeling of phrenic motoneuron dendrites (present in all animals) could not always be traced to a motoneuron cell body and cell bodies were frequently not labeled (Figure 1B).

AAV8 resulted in limited GFP expression in the cervical spinal cord (3 weeks: n = 2; 10 weeks: n = 1; data not shown); however, GFP was not detected in phrenic motoneurons. AAV9 resulted in transduction of neurons throughout the spinal cord in all treated animals (3 weeks: n = 6; 10 weeks: n = 1; data not shown), with extensive GFP expression in dendrites and soma that was not limited to the phrenic motoneuron pool. Indeed, only 1 of the 7 animals displayed GFP expression in phrenic motoneurons following AAV9-GFP treatment. In addition, AAV9 showed presumed systemic distribution following unilateral intrapleural injection, since GFP expression was evident in the contralateral cervical spinal cord (data not shown).

There was no evidence of GFP expression in the cervical spinal cord, including phrenic motoneurons, at 3 or 10 weeks after intrapleural injection with AAV2 (3 weeks: n = 6; 10 weeks: n = 1), AAV6 (3 weeks: n = 6; 10 weeks: n = 1) or rhesus-10 (3 weeks: n = 2; 10 weeks: n = 1).

In summary, intrapleural injection of AAV7, 8, and 9 all resulted in some degree of GFP expression in the cervical spinal cord. Only AAV7 resulted in consistent GFP expression in phrenic motoneurons, and was thus chosen for subsequent studies. Notably, GFP expression was not detectable in the diaphragm muscle after intrapleural delivery of AAV7-GFP (n = 6).

### FLAG Protein Expression in Ventral Horn is Evident Following Intrapleural AAV

**Figure 2:**
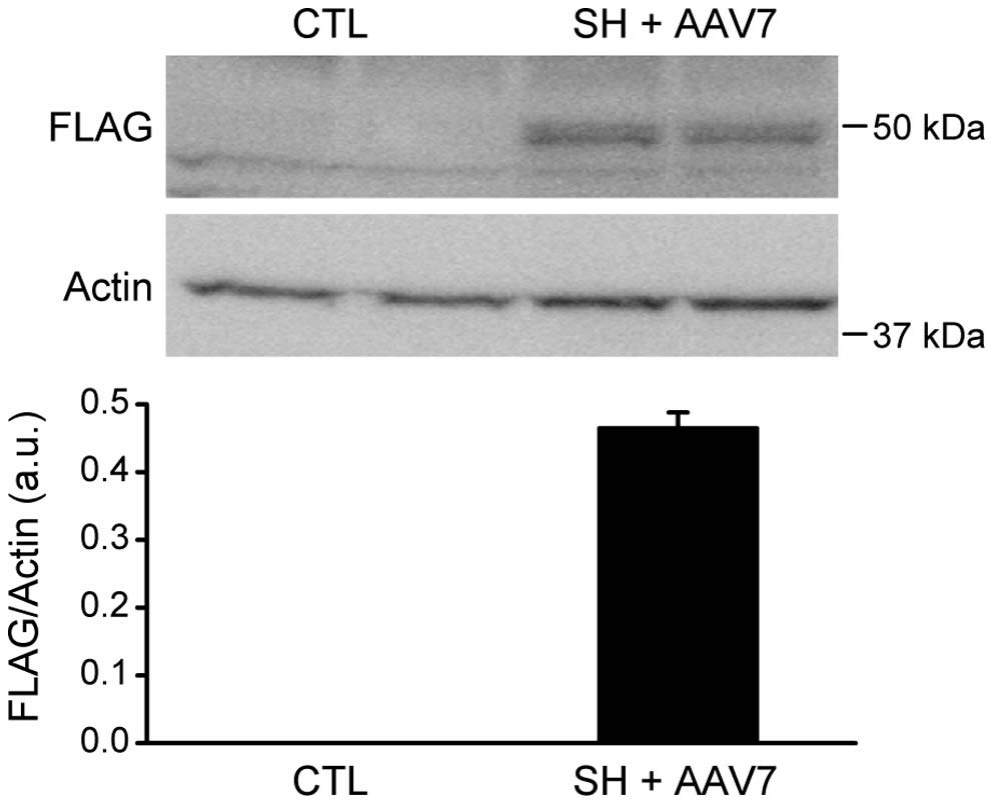
Retrograde delivery of intrapleural AAV7-TrkB.FL-FLAG to phrenic motoneurons, detected by FLAG expression in ventral horn. The cervical ventral horn region containing phrenic motoneurons was isolated and analyzed by Western blotting. *Top:* representative Western blot of FLAG (∼49 kDa) and actin (∼42 kDa) protein expression in the cervical ventral horn region from animals with C_2_ spinal cord hemisection (SH) performed at 3 weeks after intrapleural AAV7-TrkB.FL-FLAG treatment (SH+AAV7; n = 3). This is compared to that of control rats with no treatment or SH (CTL; n = 3). *Bottom*: relative expression (mean ± SE) of FLAG compared to actin. Western blot run in duplicate, left and right sides of each animal evaluated independently (bilateral AAV injection).

Delivery of AAV7-TrkB.FL-FLAG to phrenic motoneurons was evident by FLAG protein expression in the ventral horn region of the cervical spinal cord (n = 3; Figure 2). FLAG expression after AAV7 treatment was robust for all animals examined (FLAG/Actin intensity: 0.46±0.02 arbitrary units). Importantly, FLAG expression was detected at the terminal experiment 5 weeks after AAV7-TrkB.FL-FLAG treatment, consistent with the long-term expression (beyond 3 weeks) evident in immunohistochemical analyses of GFP expression. Intrapleural administration of AAV7-TrkB.FL-FLAG (n = 3) resulted in low levels of FLAG protein expression in the thoracic spinal cord, diaphragm muscle, lung, and heart (not shown). FLAG protein expression was not detected in control animals not injected with AAV7-TrkB.FL-FLAG (n = 3; Figure 2), confirming the specificity of the immunoblotting technique.

### TrkB.FL Expression in Phrenic Motoneurons

**Figure 3:**
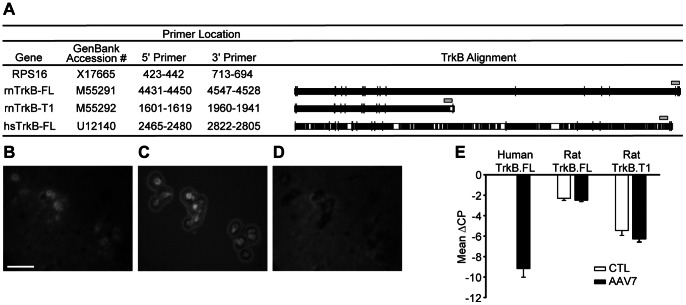
Delivery of intrapleural AAV7-TrkB.FL to phrenic motoneurons, detected by TrkB mRNA expression in microdissected motoneurons. A) Sequence alignment for TrkB receptor mRNA. White areas display regions of mismatch across all 3 mRNA transcripts. Primers used for amplification of TrkB receptor mRNA using quantitative real-time RT-PCR in microdissected phrenic motoneurons are shown above each sequence (gray box). Laser capture microdissection (LCM) was utilized to individually capture phrenic motoneurons. Representative photomicrographs display the LCM procedure. B) Retrogradely-labeled phrenic motoneurons by intrapleural injection with Alexa Fluor 488-conjugated cholera toxin subunit B. C) Selective capture of phrenic motoneurons on the LCM cap. D) Lack of Alexa 488 fluorescence reveals complete capture of selected phrenic motoneurons; Bar, 100 µm in B-D. E) Human TrkB.FL mRNA expression as well as rat TrkB.FL and rat truncated TrkB (TrkB.T1) mRNA transcripts were assessed in microdissected phrenic motoneurons 5 weeks after intrapleural injection with either saline (CTL; n = 3) or AAV7-TrkB.FL (AAV7; n = 3) in control, uninjured animals. Results of duplicate RT and real-time PCR reactions were clustered by animal after obtaining the crossing point difference (ΔCP) from RPS16, and thus reflect the relative mRNA concentration in each sample. Data are mean ± SE of ΔCP across animals.

Phrenic motoneurons were retrogradely labeled with Alexa 488-conjugated CTB, visualized by Alexa 488 fluorescence, and captured by LCM (Figure 3). Microdissection of phrenic motoneurons was confirmed by visual examination of the LCM cap as well as lack of Alexa 488 fluorescence in the remaining spinal cord tissue (Figure 3D). On average, 100–150 motoneurons were captured per animal on each side, consistent with extensive labeling of phrenic motoneurons after intrapleural injection. Since AAV7-TrkB.FL encodes human TrkB.FL, microdissected phrenic motoneurons were evaluated for human TrkB.FL mRNA expression as well as both rat TrkB.FL and truncated TrkB (TrkB.T1) mRNA transcripts using real-time quantitative RT-PCR. Primer pairs were selected such that human TrkB.FL primers did not detect rat TrkB.FL (Figure 3A). Separate melting curve peaks were observed for each primer pair, showing specificity of amplification (not shown). Samples that included no reverse transcriptase were run concomitantly and confirmed lack of genomic DNA amplification (not shown). The crossing point difference (ΔCP) from RPS16 was obtained for each transcript, reflecting the relative mRNA concentration in each sample, as previously described [6]. Human TrkB.FL mRNA was detected in AAV7-TrkB.FL treated control animals (n = 3; Figure 3E). Human TrkB.FL mRNA was not present in phrenic motoneurons from untreated control animals (n = 3). Treatment with AAV7-TrkB.FL did not change phrenic motoneuron mRNA expression of rat TrkB.FL (p = 0.44) or rat TrkB.T1 (p = 0.17).

### Proportion of Animals Displaying Functional Recovery after SH

**Figure 4:**
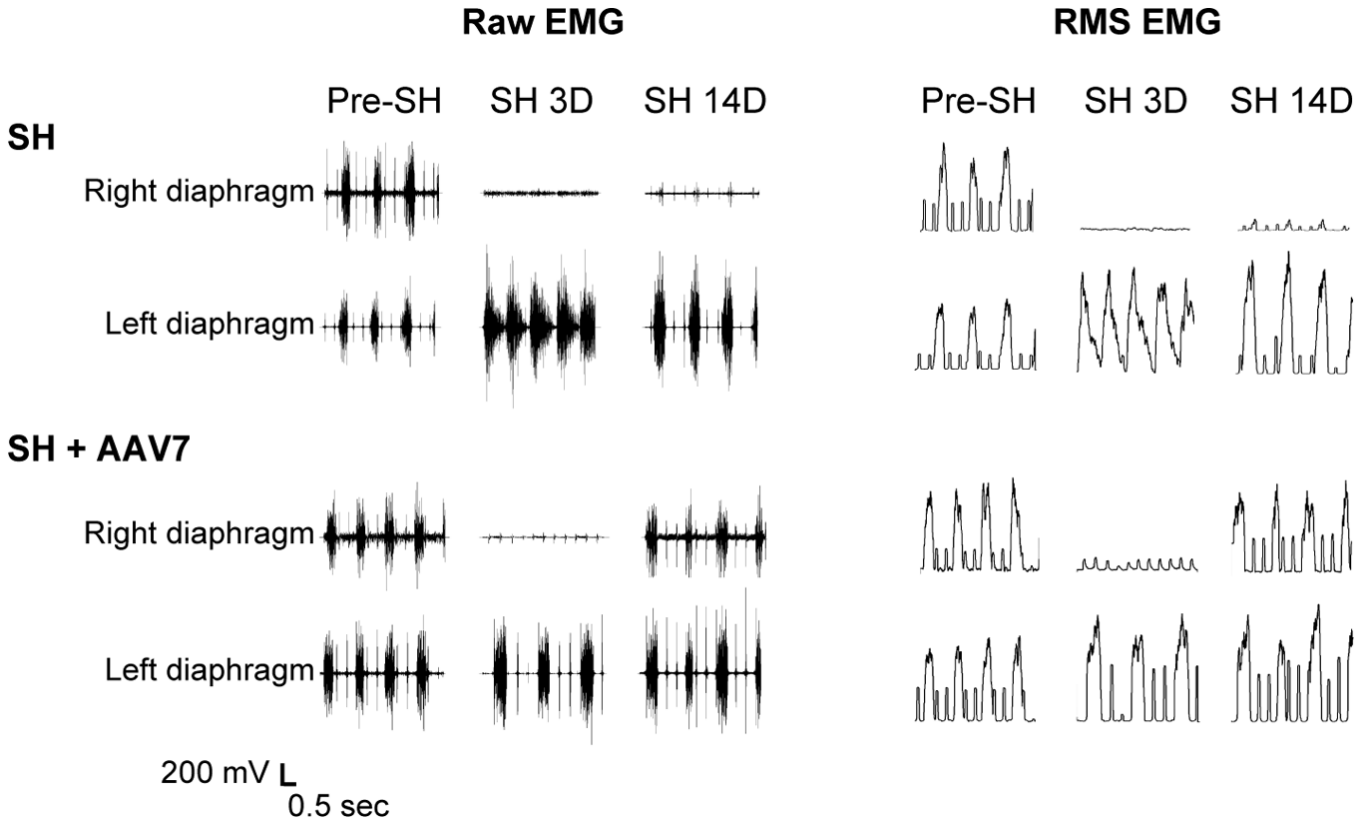
Representative raw diaphragm EMG recordings and root mean squared (RMS) EMG tracings after SH. Untreated SH animals (SH; n = 8) and SH animals treated with intrapleural AAV7-TrkB.FL (SH+AAV7; n = 11) were chronically monitored using EMG recordings obtained via implanted diaphragm electrodes during eupnea. EMG was recorded prior to (pre-SH) and at 3 days (SH 3D) and 14 days post-SH (SH 14D). Diaphragm EMG activity was absent in all animals at SH 3D, confirming complete interruption of descending ipsilateral drive to phrenic motoneurons and resulting diaphragm muscle paralysis. Both ipsilateral and contralateral diaphragm EMG activity occurred in bursts, reflecting rhythmic inspiration in the anesthetized animals. Contralateral diaphragm RMS EMG amplitude increased at SH 14D in both untreated SH and AAV7-TrkB.FL treated SH animals compared to pre-SH EMG activity. In both of the groups, there was a lack of baseline, non-rhythmic EMG activity suggestive of spasms or tonic activity in either side of the diaphragm at any time point post-SH. Intrapleural AAV9-TrkB.FL treated animals (n = 3) displayed qualitatively similar results to the untreated SH group and are not shown.

EMG electrodes were successfully implanted in all SH animals (n = 22). Animals grew as expected from an average body weight of 221±4 g prior to AAV or saline injection to 322±4 g at SH 14D. Overall, the average weight gain was ∼3 g/day in AAV7-TrkB.FL treated SH and AAV9-TrkB.FL treated SH animals. Diaphragm EMG was recorded in all SH groups at the time of surgery and at SH 3D to verify the completeness of SH surgery. No animals displayed rhythmic ipsilateral EMG activity during eupnea at SH 3D, reflecting complete interruption of descending drive to phrenic motoneurons ipsilateral to SH. Diaphragm EMG recordings were repeated during eupnea at SH 7D and SH 14D. Representative diaphragm EMG recordings and RMS EMG tracings are shown in Figure 4.

Both ipsilateral and contralateral diaphragm EMG activity occurred in bursts, reflecting rhythmic inspiration in the anesthetized animals. Contralateral diaphragm RMS EMG amplitude increased at SH 14D in both untreated SH (90±33% increase; n = 8) and AAV7-TrkB.FL treated SH animals (74±21% increase; n = 11) compared to pre-SH EMG activity (p>0.05 for across group comparison). In both of the SH groups (untreated and AAV7-TrkB.FL treated), there was a lack of baseline, non-rhythmic EMG activity suggestive of spasms or tonic activity in either side of the diaphragm at any time point post-SH (i.e., up to 14 days post-injury).

**Figure 5:**
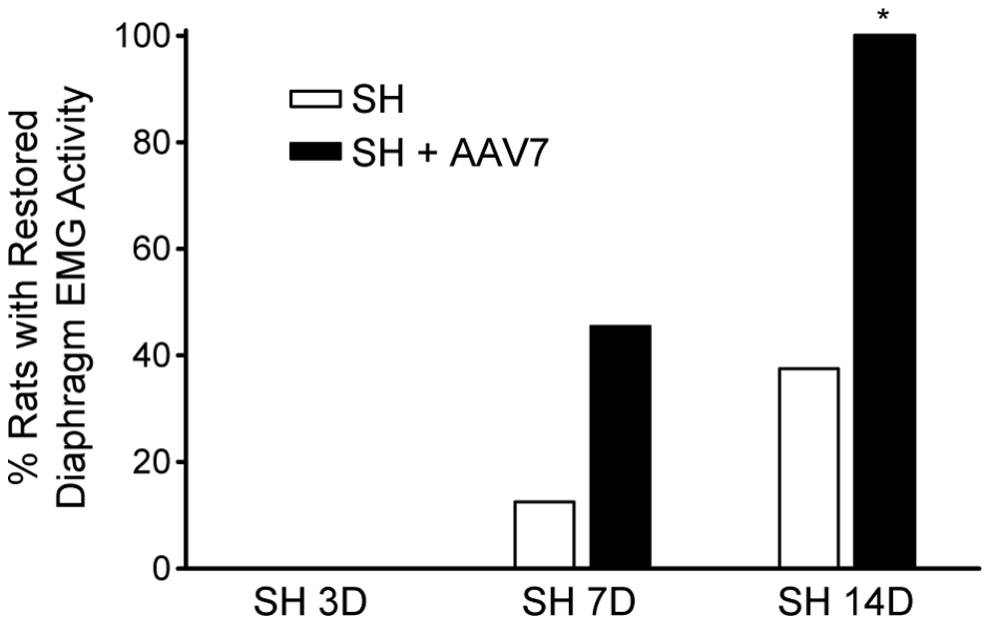
Proportion of animals displaying restored ipsilateral diaphragm EMG activity during eupnea at SH 14D. Chronic diaphragm EMG recordings were used to determine the proportion of animals displaying functional recovery after SH. Functional recovery was classified as: 1) rhythmic (i.e., in phase with the contralateral side) and periodic (i.e., occurring on at least 90% of eupneic bursts) diaphragm EMG signal; 2) diaphragm EMG signal reflecting activation of more than one motor unit; and 3) diaphragm RMS EMG amplitude at least 10% of the pre-SH amplitude. At SH 7D, five out of 11 animals with AAV7-TrkB.FL treatment (SH+AAV7) displayed functional recovery compared to one out of 8 untreated SH animals (p = 0.13). By SH 14D, all of the AAV-treated animals displayed functional recovery during eupnea compared to 3 out of 8 SH animals (*, p<0.01). None out of 3 SH animals treated with intrapleural AAV9-TrkB.FL displayed functional recovery by SH 14D (p = 0.0002 vs SH+AAV7 group).

The proportion of animals displaying functional recovery (i.e., restored ipsilateral diaphragm EMG activity) after SH was assessed in both groups. Over time after SH, a subset of untreated SH animals displayed spontaneous recovery of ipsilateral rhythmic diaphragm EMG activity during eupnea. This activity was synchronous (i.e., in phase) with the contralateral (uninjured) side, occurring in over 90% of eupneic bursts. In addition, the diaphragm EMG signal was consistently multi-unit (i.e., with multiple spike morphologies) and at least 10% of the pre-SH RMS EMG amplitude. At SH 7D, one out of 8 untreated SH animals (13%) displayed ipsilateral diaphragm EMG activity. By SH 14D, three untreated SH animals (38%) displayed functional recovery (Figure 5).

Intrapleural AAV7-TrkB.FL treatment (n = 11) increased the proportion of animals displaying ipsilateral diaphragm EMG activity after SH. At SH 7D, five animals (46%) displayed functional recovery (p = 0.13 compared to untreated SH group). By SH 14D, all of the AAV7-treated animals displayed functional recovery during eupnea (p<0.01 compared to untreated SH; Figure 5).

In contrast, intrapleural AAV9-TrkB.FL treatment did not increase recovery of ipsilateral diaphragm EMG activity after SH. None of three SH rats injected with AAV9-TrkB.FL displayed functional recovery at SH 14D (results not shown). This proportion recovered is consistent with the result of untreated SH rats (p = 0.21), but statistically less than the AAV7-TrkB.FL treatment group (p = 0.0002).

### Extent of Eupneic Diaphragm EMG Activity after SH

**Figure 6:**
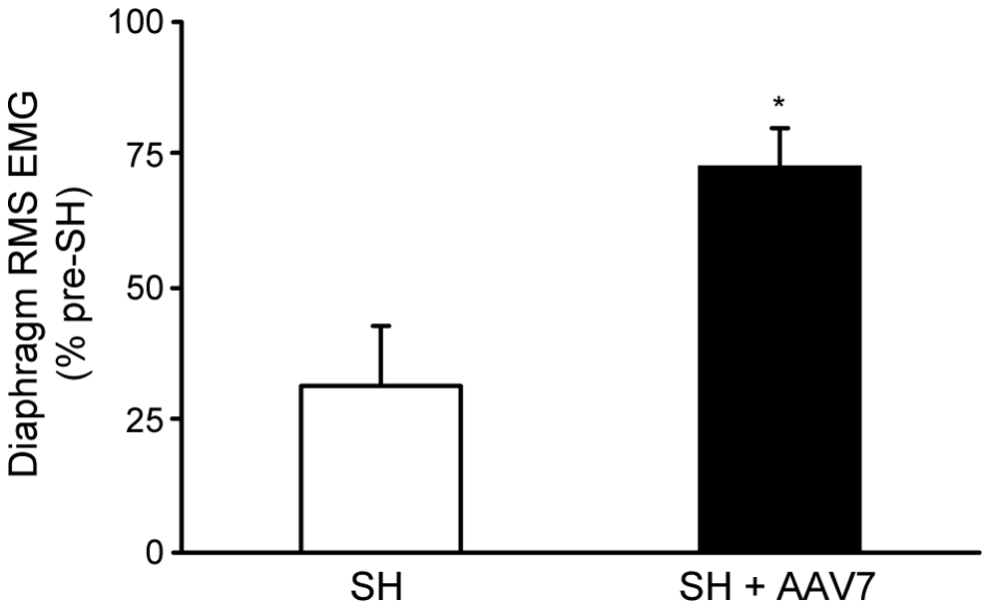
Extent of functional recovery of ipsilateral rhythmic phrenic activity at SH 14D. Diaphragm RMS EMG amplitude was measured during eupnea at various time points after SH and compared to the pre-SH RMS EMG amplitude. In those animals displaying ipsilateral diaphragm EMG activity at SH 14D, RMS EMG amplitude (mean ± SE) was reduced following SH compared to pre-injury (SH; n = 3). Treatment with AAV7-TrkB.FL increased the extent of diaphragm activity after SH (SH+AAV7; n = 11) compared to untreated SH. *, p<0.01 vs. untreated SH.

Diaphragm RMS EMG amplitude was computed to determine the extent of recovery of rhythmic diaphragm EMG activity during eupnea. Diaphragm RMS EMG amplitude was normalized to the eupneic value before SH for the same animal. Minimal variability in diaphragm RMS EMG amplitude was observed within each recording session. Treatment with AAV7-TrkB.FL significantly increased the extent of recovery after SH. At SH 7D, the 5 rats displaying functional recovery after AAV7-TrkB.FL treatment exhibited 68±17% of pre-SH eupneic RMS EMG amplitude, compared to 23% of pre-SH eupneic value in the one untreated SH animal that displayed functional recovery. At SH 14D, RMS EMG amplitude was 31±11% of the pre-SH value in the 3 untreated SH rats displaying functional recovery, and 73±7% of the pre-SH value in AAV7-TrkB.FL treated SH rats (n = 11; p<0.01 compared to the untreated SH group; Figure 6).

Respiratory patterns (respiratory rate and duty cycle) were measured from contralateral diaphragm EMG recordings during eupnea. Respiratory rate was unaffected by SH and/or AAV7-TrkB.FL treatment. In untreated SH animals (n = 8), the respiratory rate was 90±7 min^−1^ before SH and 101±6 min^−1^ at SH 14D (repeated measures ANOVA; p = 0.105). In AAV7-TrkB.FL treated SH animals (n = 11), the respiratory rate was 88±4 min^−1^ before SH and 92±4 min^−1^ at SH 14D (repeated measures ANOVA; p = 0.105). There were no differences in respiratory rate across AAV treatment groups at SH 14D (p = 0.105), consistent with a minimal effect of SH-induced unilateral diaphragm muscle paralysis on the animal’s ability to sustain eupneic ventilation.

## Discussion

The present study presents a novel, non-invasive method of targeting gene expression in phrenic motoneurons in order to promote recovery of respiratory function after cervical SCI. Recovery of ipsilateral diaphragm EMG activity is enhanced by selectively targeting delivery of TrkB receptor to phrenic motoneurons in a well-established model of incomplete SCI (SH). Motoneuron targeting was achieved by using an intrapleural delivery technique [28] combined with the transduction selectivity of AAV7. The results demonstrate that: 1) AAV7 can be retrogradely delivered to phrenic motoneurons via intrapleural injection; and 2) increasing gene expression of TrkB.FL in phrenic motoneurons via AAV7 promotes recovery of rhythmic phrenic activity ipsilateral to SH.

### Targeting Gene Expression in Phrenic Motoneurons

Injections of AAV into the pleural space have been previously used to target lung parenchyma and the diaphragm muscle [39], [40], [41]. However, previous studies injected AAV into the pleural space following thoracotomy rather than the percutaneous approach that we previously validated [28]. Indeed, this non-invasive approach has been replicated in multiple studies [6], [42], [43], [44]. Transduction of phrenic motoneurons with an intra-spinal cord (C_3_–C_4_) injection of AAV5 has been explored previously; however, transduction was not specific to the ventral horn, such that neurons in both the intermediate and ventral cervical gray matter were transduced [45]. Intra-spinal delivery of viral vectors has also been used in other models of SCI to deliver BDNF to the region of a specific motor pool [46]. Intrapleural delivery of viruses achieves optimal, selective targeting of phrenic motoneurons by matching the route of administration to the viruses’ tropism. This study provides convincing evidence for the effectiveness of using the intrapleural delivery technique to target gene expression in phrenic motoneurons.

This is also the first study to screen AAV serotypes for maximal selectivity for phrenic motoneurons. Intrapleural injection of AAV7 resulted in complete transduction selectivity for phrenic motoneurons in the cervical spinal cord. Although all animals treated intrapleurally with AAV7-GFP displayed GFP immunoreactivity only in the cervical spinal cord region containing phrenic motoneurons, commonly there was evidence of extensive dendritic labeling without identification of motoneuron soma in the same or neighboring sections. It is likely that the lack of GFP in the soma of motoneurons is the result of GFP leaking after paraformaldehyde fixation, as seemingly happens with eGFP expression (Figure 1B). Future studies could use a farnesylated GFP that would be expected to remain membrane-bound, and this modification of the technique may permit better cellular localization of AAV7 transduction. Regardless, we were able to document that phrenic motoneuron transduction efficiency was at least 11% (98/884 motoneurons in 6 rats). This estimate compares very favorably with previous studies of motoneuron transduction by AAV using alternative delivery routes. For example, Towne et al. reported AAV transduction efficiency of cervical, thoracic and lumbar motoneurons as 3–4% following intravenous delivery in 3 mice [47].

In this study, transduction selectivity for phrenic motoneurons was the primary criterion used in screening AAV serotypes. For example, intrapleural AAV9-GFP treatment displayed low selectivity for phrenic motoneurons, resulting in both ipsilateral and contralateral GFP expression in presumptive spinal neurons (not selective to phrenic motoneurons) in all treated animals, and GFP expression in phrenic motoneurons was evident in only 1 out of 7 treated animals. In support of the importance of selective transduction of phrenic motoneurons, AAV9-TrkB.FL did not increase recovery of ipsilateral diaphragm EMG activity after SH. As such, AAV9-TrkB.FL suggests that intrapleural viral treatment is itself insufficient to enhance recovery. It is unlikely that a non-specific effect (e.g., an inflammatory response) contributed to the AAV7-mediated enhancement of functional recovery after SH. Indeed, transduction of motoneurons via intrathecal injection of various AAV serotypes has not revealed an inflammatory reaction beyond 10 days and up to 15 weeks after treatment [48], [49]. In the present study SH was performed 3 weeks after intrapleural AAV7-TrkB.FL injection, and thus we do not expect that a non-specific inflammatory response contributed to functional recovery in this group.

Multiple converging results document selective transduction of phrenic motoneurons in the cervical spinal cord by AAV7 transduction. First, GFP expression was identified in retrogradely-labeled phrenic motoneurons. FLAG protein expression (a product of the TrkB.FL-FLAG construct) was present in the cervical ventral horn region containing phrenic motoneurons. In addition, AAV7-mediated delivery of TrkB.FL to phrenic motoneurons was confirmed by measuring human TrkB.FL mRNA expression in microdissected phrenic motoneurons. Human TrkB.FL mRNA was only expressed in AAV7-TrkB.FL treated animals, validating the specificity of the quantitative mRNA technique. Of note, AAV7-TrkB.FL-mediated transduction of phrenic motoneurons resulted in human TrkB.FL expression in phrenic motoneurons without a change in expression of endogenous TrkB.FL and TrkB.T1 mRNA in the rat. Possible differences in PCR efficiency between the rat and human TrkB.FL gene products limit measurements of total TrkB.FL expression in AAV7-treated animals, and accordingly, we could not confirm increased overall TrkB.FL in phrenic motoneurons. In this regard, the LCM procedure employed in the present study did not selectively capture only motoneurons with evidence of AAV7 transduction (e.g., displaying FLAG immunoreactivity). Future studies could stratify mRNA expression in phrenic motoneurons based on AAV7 transduction in order to assess the relative expression of exogenous TrkB.FL compared to endogenous TrkB.FL. Regardless, selective transduction of phrenic motoneurons with TrkB.FL was sufficient to promote functional recovery of rhythmic phrenic activity following cervical SCI.

The intrapleural injection technique can also be useful for targeting motoneurons in other regions of the spinal cord. For instance, thoracic motoneurons likely innervating intercostal muscles were labeled retrogradely by intrapleural CTB [28]. Of note, low levels of FLAG protein expression were detected in the thoracic spinal cord, diaphragm muscle, liver, and heart, suggesting that AAV7 was highly selective for phrenic motoneurons in the cervical spinal cord, but also resulted in limited delivery to other tissues. It is highly unlikely that expression of TrkB.FL in any of these other tissues contributed to the recovery of rhythmic diaphragm EMG activity ipsilateral to SH.

### Mechanism for Effects of TrkB.FL on Functional Recovery

Targeted delivery of TrkB.FL to phrenic motoneurons increased both the proportion of animals displaying ipsilateral diaphragm EMG activity and the extent of recovery compared to untreated SH animals. A change in the balance between full-length and truncated TrkB receptors in phrenic motoneurons may represent a major mechanism by which neurotrophin signaling can be modulated and ipsilateral diaphragm EMG activity restored. Only TrkB.FL is capable of signaling via kinase activity and phosphorylation, while truncated TrkB isoforms may modulate TrkB signaling [50], [51], [52], [53]. Intrapleural AAV7-TrkB.FL increased TrkB.FL expression (of the human homolog) without a change in rat TrkB receptor isoforms in phrenic motoneurons. Much work has documented the high sequence alignment between human and rat TrkB [54], [55], such that it seems reasonable to expect that increasing TrkB expression (human or rat) will result in similar intracellular signaling effects. Although the relative role of TrkB.FL and TrkB.T1 in functional recovery of rhythmic phrenic activity post-SH is not known, the results of the present study strongly indicate that increasing TrkB.FL signaling in phrenic motoneurons is sufficient to promote recovery of ipsilateral diaphragm EMG activity.

The main excitatory synaptic inputs to phrenic motoneurons are glutamatergic [56], [57] or serotonergic [9], [37], [58], [59]. Following SH, expression of NMDA in phrenic motoneurons increased and AMPA expression decreased [6], [60]. In addition, phrenic motoneuron expression of 5-HTR2a (and possibly 5-HTR2c) receptors increased [6], [24]. The SH model is a well-validated model of cervical SCI in which spontaneous recovery of ipsilateral diaphragm activity is generally thought to result from strengthening of spared synaptic inputs to phrenic motoneurons [9], [10], [11], [12], [13], [14], although the relative importance of glutamatergic and serotonergic transmission to functional recovery is still unclear. Changes in glutamatergic and serotonergic neurotransmission in the region of the phrenic motor nucleus generally correspond with the timing of spontaneous recovery of rhythmic phrenic activity [6], [24], [60], and likely contribute to functional recovery by increasing motoneuron excitability [61]. The intrapleural AAV7-mediated transduction of phrenic motoneurons provides a useful tool to directly examine the interaction between BDNF/TrkB.FL signaling and glutamatergic or serotonergic neurotransmission at phrenic motoneurons.

Expression of neurotrophins and their receptors changes following SCI [62], [63], [64], [65], but whether expression of neurotrophins or their receptors increases at motoneurons following SH is not clear. Although gene expression in phrenic motoneurons has not been specifically explored after SH, following a midthoracic SCI in rats there is a generalized increase in truncated TrkB receptor mRNA expression in neurons and surrounding glia [63], [64], including levels below the injury site (i.e., corresponding to the phrenic motor nucleus). In the present study, BDNF signaling via full-length TrkB in phrenic motoneurons was likely enhanced by AAV7-TrkB.FL-mediated transduction. Several studies show increased neuron survival and axonal sprouting by BDNF treatment following SCI [27], either via intrathecal infusion [25], [66], [67], [68], intraspinal viral transduction [46], [69], or stem cell transplantation [70], [71]. In addition, neurotrophins such as BDNF can upregulate TrkB receptor expression through a positive feedback effect [72], [73]. Future studies could address the combination of AAV-mediated motoneuron transduction with exogenous neurotrophin treatment to limit adverse effects related to neurotrophin treatment [25], [26] and maximize therapeutic benefits. In this sense, novel small molecular TrkB ligands such as 7,8-dihydroxyflavone [74], [75], which cross the blood brain barrier [76], may help overcome issues related to neurotrophin penetration and bioavailability.

### Therapeutic Benefit of TrkB.FL

This study found that functional recovery of rhythmic phrenic activity after SH is enhanced when the animals are injured at a time when they display increased expression of TrkB.FL in phrenic motoneurons. Importantly, this study does not demonstrate that TrkB.FL can be used therapeutically to enhance recovery after SCI, but rather provides exciting proof-of-concept information regarding the importance of selectively targeting TrkB.FL signaling to phrenic motoneurons in promoting functional recovery after SH. Future studies evaluating the effect of treatments initiated at some time following injury (a more clinically relevant scenario where treatment usually starts after an acute phase of spinal shock) will be important in determining a therapeutic window. Regardless, this study provides direct evidence that increased TrkB.FL expression in phrenic motoneurons is sufficient to enhance recovery of ipsilateral rhythmic phrenic activity after SH, indicating that selectively targeting gene expression in spared motoneurons below the level of incomplete SCI may promote functional recovery.
